# Restoring neuropetide Y levels in the hypothalamus ameliorates premature aging phenotype in mice

**DOI:** 10.1007/s11357-025-01574-0

**Published:** 2025-02-27

**Authors:** Marisa Ferreira-Marques, Sara Carmo-Silva, Joana Pereira, Mariana Botelho, Clévio Nóbrega, Carlos López‐Otín, Luís Pereira de Almeida, Célia A. Aveleira, Cláudia Cavadas

**Affiliations:** 1 CNC-UC, Center for Neuroscience and Cell Biology, University of Coimbra, Coimbra, Portugal; 2 CIBB - Center for Innovative Biomedicine and Biotechnology, University of Coimbra, Coimbra, Portugal; 3 H&TRC – Health and Technology Research Center, Coimbra Health School, Polytechnic University of Coimbra, Coimbra, Portugal; 4 Citechcare—Center for Innovative Care and Health Technology, Polytechnic University of Leiria, Leiria, Portugal; 5 Algarve Biomedical Center Research Institute (ABC-RI), University of Algarve, Faro, Portugal; 6 Faculty of Medicine and Biomedical Sciences (FMCB), University of Algarve, Faro, Portugal; 7 Departamento de Bioquímica y Biología Molecular, Facultad de Medicina, Instituto Universitario de Oncología, Universidad de Oviedo, Oviedo, Spain; 8 Centre de Recherche Des Cordeliers, Inserm U1138, Sorbonne Université, Paris, France; 9 Facultad de Ciencias de La Vida y La Naturaleza, Universidad Nebrija, Madrid, Spain; 10 MIA-Portugal - Multidisciplinar Institute of Ageing, University of Coimbra, Coimbra, Portugal; 11 Faculty of Pharmacy, University of Coimbra, Coimbra, Portugal

**Keywords:** Biology of aging, Brain aging, Hypothalamus, Neuropeptide Y

## Abstract

The hypothalamus has been recognized as a regulator of whole-body aging. Neuropeptide Y (NPY), highly abundant in the central nervous system and produced by the hypothalamus, enhances autophagy in this brain region and mediates autophagy triggered by caloric restriction, suggesting a potential role as a caloric restriction mimetic and an aging regulator. Considering that hypothalamic NPY levels decline during aging, we investigated if reestablishment of NPY levels mitigate aging phenotype, using a mouse model of premature aging – *Zmpste24*^−/−^ mouse. The results show that reestablishing hypothalamic NPY levels delayed aging-associated features, including lipodystrophy, alopecia, and memory. Moreover, these results suggest that strategies that promote maintenance of hypothalamic NPY levels might be relevant to counteract aging progression and age-related deteriorations.

**Supplementary Information:**

The online version contains supplementary material available at 10.1007/s11357-025-01574-0.

## Introduction

The hypothalamus is crucial for the neuroendocrine interaction between the central nervous system and the periphery, regulating functions like reproduction, sleep, and energy balance [1]. This regulatory role relies on specialized neuronal circuits organized into different nuclei within this brain region [2–4]. These inputs coordinate behavioral and autonomic functions involved in food intake, energy expenditure, and neuroendocrine responses [3, 5]. Several studies have shown that age-associated decline in hypothalamic function is a key factor in the development of whole-body aging [6–11]. The hypothalamic–pituitary–growth hormone (GH) axis regulates several physiological aspects related to longevity; further supporting this brain region as may be determinant for lifespan [12, 13]. The age-dependent decline of hypothalamic function and its consequent effect on neuroendocrine dysfunction during aging might be linked to several mechanisms including defective autophagy, impaired neurogenesis, microglia activation, increased expression of inflammatory cytokines, and activation of NF-κB signaling [9, 14–16]. The inflammatory environment in the hypothalamus is also a relevant contributor to whole-body age-related dysfunctions, since suppressing the hypothalamic IKKβ/NF-κB pathway activation delayed age-related changes and increased the lifespan of mice [9]. Considering that the hypothalamus may counteract systemic age-associated functional changes and promote longevity in mammals [7–9], targeting this brain region is a promising therapeutic strategy to block or delay the deteriorations that occur in whole-body aging. However, the cellular and molecular mechanisms that occur in the aging hypothalamus remain largely unknown.

The hypothalamic neuropeptide Y (NPY) responds to low energy availability and represents the primary hunger signal during caloric restriction [17]. Numerous studies suggest a putative relevant role of hypothalamic NPY in delaying aging: 1) hypothalamic NPY levels decline with age [18]; 2) caloric restriction, a strategy known to extend lifespan, increases NPY levels in the arcuate nucleus (ARC) of the hypothalamus [19]; 3) transgenic mice overexpressing hypothalamic NPY show stress resistance and increased lifespan [20], and caloric restriction does not increase lifespan in NPY knockout mice [21]. Our previous findings further support a relevant role of hypothalamic NPY in delaying age-related features. We showed that NPY induces autophagy and mediates caloric restriction-induced autophagy in hypothalamic rodent neurons [22]. Since autophagy decreases with age [23] and the restoration of this cellular process has been correlated with increased lifespan [24], suggests that boosting NPY levels in the hypothalamus may, directly or indirectly, counteract aging.

Aging studies in laboratory settings require animal models. The Zmpste24 (Z24) KO mice lack a metalloproteinase involved in the maturation of lamin A, an essential component of the nuclear envelope [25]. These mice exhibit a premature aging phenotype, with multiple histopathological defects on skin, bone, cardiovascular tissues, and skeletal muscles that phenocopy human accelerated aging processes, leading to premature death [26]. Therefore, Z24-KO mice are a suitable mouse model of aging appropriate for studying aging mechanisms.

Considering the pivotal role of the hypothalamus in whole-body aging and the age-associated decline of NPY levels, adjusting hypothalamic NPY could prevent age-related hypothalamic dysfunction, impacting aging and lifespan. Building on our previous studies, we hypothesized that reestablishing hypothalamic NPY levels might deaccelerate aging. Specifically, in the present study we investigate whether the increase of hypothalamic NPY levels specifically in the hypothalamus could counteract premature aging in a mouse model of premature human aging—the *Zmpste24*^−/−^ mouse [25].

## Materials and methods

### Animals

*Zmpste24*^−/−^ or Zmpste24-KO (Z24-KO) mice (C57BL/6 background) were generated and genotyped in the laboratory of Carlos López-Otín (University of Oviedo, Spain) as previously described [25]. Two-months-old male and female Z24-KO mice were randomly divided into two groups: Z24-KO mice (vehicle-treated Z24-KO mice; 4 males and 3 females) and hypothalamic NPY-overexpressing Z24-KO mice (NPY-treated Z24-KO; 6 males and 6 females). Adult male C57BL/6 mice (wild-type mice) aged 5–6 months were obtained from Charles River, Spain. Mice were housed in pairs *per* cage, under a 12:12-h light/dark cycle, with controlled temperature and humidity and provided to the same quantity of normal standard chow diet (4RF25 top certificate, from Mucedola Srl). All experimental work was approved by the CNC-UC Animal Welfare Body (ORBEA 329 and DGAV 009428) and performed following the European Community directive for the care and use of laboratory animals (86/609/EEC) and the Portuguese law for the care and use of experimental animals (Decree-law 113–2013). The animals were housed in the licensed animal facility of CNC-UC International Animal Welfare Assurance (number 520.000.000.2006). Animal experimentation was performed by credited and trained investigators, as required by the Portuguese authorities.

### Sustained Increase of Neuropeptide Y levels in the mouse hypothalamic arcuate nucleus

To manipulate the endogenous expression of NPY, recombinant adeno-associated virus (AAV)-NPY vectors were injected through bilateral stereotaxic injection in the arcuate nucleus (ARC) of the hypothalamus (AAV-NPY Z24-KO mice), as previously described [27–29]. Recombinant AAV particles were generated as previously, using AAV-1/2 chimerical capsids containing recombinant plasmids with NPY cDNA under a neuronal-specific promoter, the human synapsin promoter, to induce constitutive NPY overexpression in hypothalamic ARC of mice [29]. The human synapsin promoter in the viral vector ensures that only mature neurons will express the transgene. This proximal region of the synapsin promoter is highly conserved between mouse and human [27, 28]. Mice were anesthetized with an intraperitoneal injection of ketamine/xylazine (100 mg/kg and 10 mg/kg, respectively) and placed on a stereotaxic frame. The ARC coordinates were defined by using the Paxino’s Mouse Brain Atlas and bilateral injections were performed into the ARC at 0.5 mm lateral to the midline, 1.58 mm posterior to the bregma, and − 5.8 mm ventral to the brain surface. The NPY-treated Z24-KO mice received 3.6 × 10^9^ v.g. *per* side of AAV-hSyn-NPY, in a final volume of 1.5 μL *per* side. The Z24-KO mice received saline solution (0.9% NaCl) in the exact same conditions. Injection was performed at a rate of 0.5 μL/min with a 10 μL-Hamilton syringe attached to an automatic Pump Controller (WPI), and the needle was kept in place for 5 min to minimize backflow. Mice were allowed to recover for 2 days. The experimental endpoint of this experiment was defined at 4 months following AAV injection. As NPY is a potent orexigenic neuropeptide, to avoid significant weight changes, NPY-treated Z24-KO mice were pair-fed, receiving the same amount of food that saline-treated Z24-KO mice ate daily (approximately 4–5 g/day). The body weight of each mouse was assessed every other day for weight control. Fur loss was evaluated biweekly and scored on a scale of 0 to 3 (0 for absent, 1 for mild, 2 for moderate, and 3 for marked fur loss).

### Open field test

For the assessment of mice locomotor horizontal activity and anxiety-like behaviors, the open field test was conducted at 30, 60, 90 and 120 days following stereotaxic injection. Prior to the experiment, mice were acclimated to the test room for a 12-h period. During the test, each mouse was individually placed in the center of a 50 × 50 cm arena with 50 cm high walls, and their movement activity was recorded for 40 min using the Acti-Track System (Panlab, Barcelona, Spain). The activity tracing of the two zones of the box and the mean values for total distance travelled and velocity were analyzed [30].

### Y-maze test

For the assessment of spatial working and reference memory, the Y-maze test was conducted at 30, 60, 90 and 120 days following stereotaxic injection. Prior to the experiment, mice were acclimated to the test room for a 12-h period. During the test, mice were gently introduced to the central point of y-maze test, consisted of a Y-shaped maze with three arms, each measuring 38 cm in length, 7.5 cm in width, and separated by 120-degree angles. Subsequently, mice were given a 10-min period to freely explore the three arms while being recorded on video. The number of entries into each arm and the time spent in each arm were analysed. These data were then utilized to calculate the percentage of alternation, a measure used to evaluate mice to explore different arms in a non-repetitive manner.

### Tissue and blood collection

Animals were euthanized 4 months after stereotaxic injections using a sodium pentobarbital overdose. Each group of animals was randomly selected for either hypothalamic tissue collection for protein extraction or whole brain removal for immunohistochemistry experiments. For biochemical analyses, blood was collected, and the serum was separated by centrifugation at 2,000 × *g* for 15 min. Serum samples were then stored at −20 °C until use. Hypothalami were individually collected and stored at −80 °C until use. For immunohistochemistry, animals were intracardially perfused with a 4% (wt/vol) paraformaldehyde/PBS fixative solution. After decapitation, the brains were removed and immersed in a 25% (wt/vol) sucrose solution in 0.1 M PBS at 4 °C for 48 h for cryoprotection. The brains were then stored at −80 °C until further use. Regarding the peripheral organs (skin, liver, heart and kidney), they were cut and divided. One part of each organ was kept at −80 °C for protein analyses, while the other part was kept in a 10% neutral buffered formalin solution for 48 h to prepare them for histological processing.

### Western blotting

Tissues were lysed on ice in RIPA (radio-immunoprecipitation assay) buffer [50 mM Tris·HCl, pH 7.4; 150 mM NaCl; 5 mM EDTA; 1% Triton X-100; 0.5% deoxycholate; 0.1% SDS; 200 μM phenylmethylsulphonyl fluoride; 1 mM DTT, 1 mM Na_3_VO_4_; 10 mM NaF], supplemented with a mini protease inhibitor mixture tablet (Roche). Lysates were incubated for 15 min at 4 °C, and the insoluble material was pelleted by centrifugation for 10 min at 16,000 × g and 4 °C. The protein concentration of each sample was determined using the bicinchoninic acid protein assay (Pierce Biotechnology). Samples were then boiled in SDS sample buffer, run on a 4%−20% polyacrylamide gel (Bio-Rad), and transferred to an Immobilon-P polyvinylidene fluoride (PVDF) membrane (Millipore). The membrane was blocked with 5% BSA in TBST (TBS with 0.1% Tween-20) at room temperature for 1 h and then probed with primary antibodies (all at a dilution of 1:1,000) overnight in 1X TBST with 5% BSA. The primary antibodies used were as follows: mouse anti-NeuN (Neuronal Nuclear Protein) (Chemicon, Merck Millipore), rabbit anti-GFAP (Glial Fibrillary Acidic Protein) (Dako), mouse anti-phospho-IκBα (Cell Signaling Techonology), mouse anti-Tau (Invitrogen Antibodies), mouse anti-PCNA (Santa Cruz Biotechnology), mouse anti-phospho-p53 (Cell Signaling Techonology), rabbit anti-KRT1 (BioLegend), rabbit anti-SQSTM1 (Cell Signaling Techonology) and rabbit anti-LC3B (Cell Signaling Techonology). Protein immunoreactive bands were visualized by chemiluminescence with the ECL substrate (Amershan, Cytiva) in a ImageQuant 8000 Imaging System (Amershan, Cytiva). The optical density of the bands was quantified using the Image Lab Software 6.0.1 (Bio-Rad). The results were normalized to the amount of β-Actin (MilliporeSigma) or β-Tubulin (MerckMillipore) (all at a dilution of 5,000) and are expressed as the relative amount compared with Z24-KO.

### Immunohistochemistry

For immunohistochemistry, brains were cut in 25 μm coronal sections using a cryostat-microtome (Leica CM3050S, Leica Microsystems Nussloch GmbH). Slices were collected and stored in 48-well plates, free floating in 0.1 M PBS supplemented with 0.12 μmol/L sodium azide. The plates were stored at 4 ºC until immunohistochemical processing. Briefly, brain sections were washed twice with PBS and blocked and permeabilized in PBS with 10% GS and 0.3% (v/v) TX-100, for one hour at room temperature (21–25 ºC). Brain slices were then incubated with a polyclonal rabbit anti-NPY antibody (1:6,000; Sigma-Aldrich), monoclonal mouse anti-MAP2 (Microtubule Associated Protein 2) antibody (1:500; Sigma-Aldrich), monoclonal mouse anti-NeuN (Neuronal Nuclear Protein) antibody (1:500; Chemicon, MerckMillipore), polyclonal rabbit anti-GFAP (Glial Fibrillary Acidic Protein) antibody (1:1,000, Dako), polyclonal rabbit anti-Iba1 (Ionized Calcium-Binding Adapter Molecule) antibody (1:315; WAKO) in blocking solution, overnight at 4 °C. The sections were then washed in PBS and incubated with goat anti-rabbit Alexa-Fluor 594-, goat anti-mouse Alexa Fluor 594-, or goat anti-rabbit Alexa Fluor 488-conjugated secondary antibodies, for two hours at RT. Nuclei were counterstained with Hoechst 33342 (2 μg/mL; Invitrogen). After incubation, brain sections were washed in PBS and mounted in slides with Mowiol® mounting medium (Sigma-Aldrich). In the end of the procedure, slides were analysed on a Zeiss Axiovert fluorescence microscope (Carl Zeiss) or Axio Imager Z2 (Carl Zeiss) and Axio Observer inverted microscope (Carl Zeiss).

### Histological analysis

After fixation in formalin, tissue samples were cut into small fragments then submitted to several steps for paraffin blocks embedding: 1 h at ethanol 70%; followed by two series of 95% ethanol for 45 min each, two series of 100% ethanol for 1 h each, two series of xylene for 1 h each, and finally two series of paraffin for 1 h each. At the end of this process, the tissue samples were embedded in paraffin blocks. Paraffin blocks were sectioned using a microtome (HM325, Thermo Fisher Scientific). The 3–5 μm thickness sections were placed into microscopy slides until use. Haematoxylin–Eosin staining was performed following the manufacturer’s instructions. After staining, sections were mounted in slides with Richard-Allan Scientific Mounting Medium (HM325, Thermo Fisher Scientific) and analysed on a Zeiss Axio Imager Z2 microscope (Zeiss). The nuclei were stained blue and the cytoplasm red, to detect structural alterations in the tissue. This procedure was performed for skin, liver, heart, and kidney. Images were analysed with Fiji Software. The analysis was conducted by an independent researcher blinded to the experimental groups.

### Determination of NPY content

Serum samples were assayed for NPY concentration using an NPY EIA kit (RayBiotech), following the manufacturer’s instructions.

### Statistical analysis

Results are expressed as mean ± standard error of the mean (SEM). Statistical differences between groups were assessed using appropriate statistical tests based on the number of variables and groups compared. For comparisons between two groups wild-type and Z24-KO or Z24-KO and AAV-NPY Z24-KO, an unpaired Student’s *t*-test with one-tailed *p* value was used. For comparisons involving more than two groups or two independent variables (days after surgery and treatment groups) a Two-way ANOVA was performed. The categorical variables were compared using the Chi-square test. All statistical analyses were conducted using Prism 8.42 (GraphPad Software). Statistical significance was set at *p* < 0.05. Specific statistical significances can be found in the figure legends.

## Results

### Z24-KO mice exhibit lower levels of NPY in the hypothalamic arcuate nucleus

**Fig. 1:**
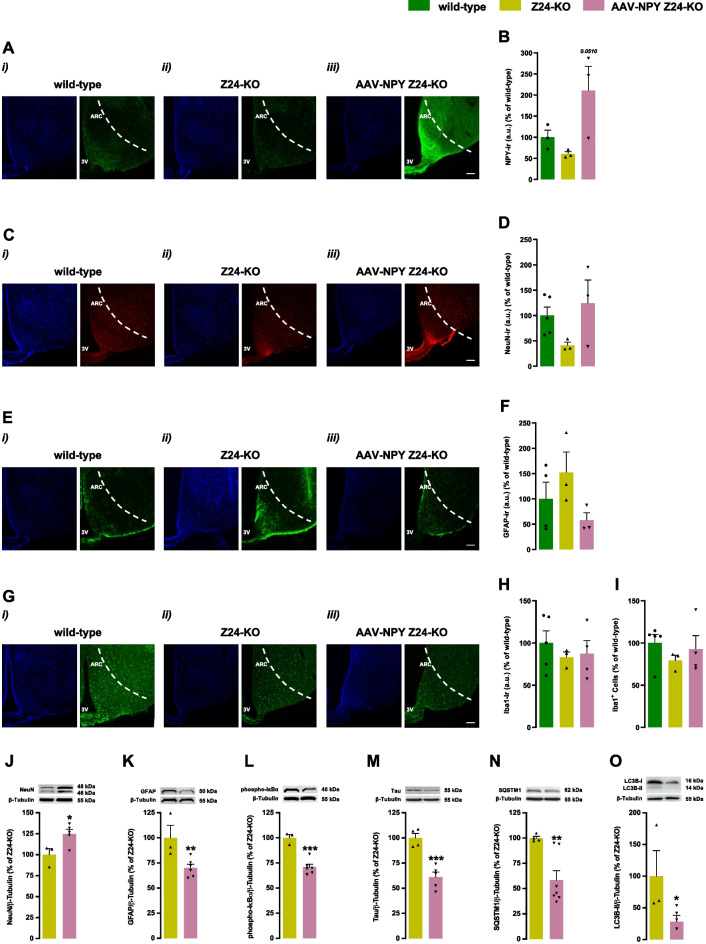
Z24-deficient mice display lower levels of NPY in the hypothalamic arcuate nucleus. (**A** and **B**) Z24-KO mice show decreased hypothalamic ARC NPY levels, restored upon NPY-overexpression. Representative NPY-immunoreactivity images in the hypothalamus ARC (**A**). Quantification of NPY-immunoreactivity across the anterior–posterior length of the ARC (**B**). **C** and **D** NeuN levels are decreased in Z24-KO mice hypothalamic ARC, and NPY-overexpression restores its levels. Representative NeuN-immunoreactivity images in the hypothalamus (**C**). Quantification of NeuN-immunoreactivity through the anterior–posterior length of the ARC (**D**). **E** and **F** GFAP levels increased in the Z24-KO mouse hypothalamic ARC, with restoration upon NPY-overexpression. Representative images display GFAP-immunoreactivity in the hypothalamus (**E**). Quantification of GFAP-immunoreactivity through the anterior–posterior length of the mouse ARC (**F**). **G**, **H**, and **I** Iba1 levels decrease in Z24-KO hypothalamic ARC, while NPY-overexpression restores them. Representative Iba1-immunoreactivity images in the hypothalamus (**G**). Quantification of Iba1-immunoreactivity through the anterior–posterior length of the ARC (**H**). Quantification of the number of Iba1-positive cell count in hypothalamic ARC (**I**). **J**, **K**, **L**, **M** and **N** The hypothalamus of AAV-NPY Z24-KO mice shows higher NeuN, and lower GFAP, phospho-IκBα, Tau, SQSTM1 and LC3B-II *vs*. Z24-KO mice. Whole hypothalamic lysates collected at the experiment's endpoint (four months post-AAV-NPY injection) were subjected to Western blotting NeuN (**J**), GFAP (**K**), phospho-IκBα (**L**), Tau (**M**), SQSTM1 (**N**) and LC3B-II (**O**) with β-Tubulin (loading control) immunoreactivity. Representative Western blots above the respective graph. Results presented as the mean ± SEM. *p < 0.05, **p < 0.01, ***p < 0.001, via Student’s *t-*test. Scale bar, 100 μm. *N* = 3–7 *per* experimental group. Z24-KO = Zmpste24-KO; AAV = Adeno-Associated Virus; NPY = Neuropeptide Y; 3 V = third ventricle; ARC = Arcuate Nucleus

We hypothesized that Z24-KO mice, with a premature aging phenotype, might recapitulate alterations within the hypothalamus in natural aged rodents [31–33]. Initially, we compared NPY immunoreactivity in the hypothalamic ARC of Z24-KO mice with age-matched wild-type mice. Z24-KO mice showed lower levels of NPY immunoreactivity, compared to age-matched wild-type mice (Fig. 1Ai-Aii and B). Moreover, in Z24-KO mice, we observed lower levels of the neuronal marker (NeuN) in the ARC of Z24-KO, suggestive of fewer hypothalamic neurons (Fig. 1Ci-Cii, D, and J). Aging is also characterized by neuroinflammation, characterized by elevated astrocytic activity and gliosis in the hypothalamus [9]. Z24-KO mice present increased GFAP levels, a gliosis marker (Fig. 1Ei-Eii, F, and K), suggesting neuroinflammation in the hypothalamus of Z24-KO mice. Interestingly, the immunoreactivity of the microglia marker ionized calcium-binding adaptor molecule 1 (Iba1) was lower in Z24-KO mice (Fig. 1Gi-Gii, H, and I), suggesting a compromised immune response capacity in these mice.

Considering the age-related decrease in NPY hypothalamic levels in Z24-KO mice, we hypothesized that reestablishing the NPY levels in the hypothalamus could delay the aging phenotype of Z24-KO mice. Using an injection of AAV encoding for NPY (AAV-NPY) in the hypothalamus, we achieved a sustained upregulation of NPY levels during four-months post-injection (Fig. 1Aii-Aiii and B). Importantly, this induction of NPY expression was restricted to the ARC (Supplementary Fig. S1).

Z24-KO mice injected with AAV-NPY mice (AAV-NPY Z24-KO) show relevant alterations within the brain to similar levels of age-matched wild-type mice, namely reestablishment of NeuN levels (Fig. 1Cii-Ciii, D, and J) and a decrease of GFAP, (Fig. 1Eii-Eiii, F, and K). However, no significant changes were observed in microglial immunoreactivity of AAV-NPY 24KO mice (Fig. 1Gii-Gii, H, and I). Moreover, AAV-NPY Z24-KO mice show lower levels of phosphor-Iκβα and Tau, compared to non-injected Z24-KO, indicative of decreased NF-κB activation and Tau pathology, respectively (Fig. 1L and M). Autophagy plays a critical role in regulating longevity [34]. Moreover, we previously showed that NPY increases autophagic flux in rodent hypothalamic neurons [22]. Accordingly, we evaluated a relevant autophagy markers, LC3B-II and SQSTM1 proteins. Mice overexpressing hypothalamic NPY exhibited decreased levels of SQSTM1 (Fig. 1N) and LC3B-II (Fig. 1O) in the hypothalamus, compared with Z24-KO mice. The reduction in LC3B-II and SQSTM1 protein levels suggests faster protein degradation in these mice, consistent with an increase of the autophagic flux in the hypothalamus.

Collectively, these findings suggest that Z24-KO mice show relevant aging alterations in neuronal structure, astrocytic activity, innate immune response, and inflammation within the hypothalamic ARC. Notably, reestablishment of NPY levels in the hypothalamus mitigated these age-related changes, highlighting the potential benefits of NPY by preventing neuronal integrity and health and preventing inflammatory responses in the hypothalamus.

### Hypothalamic NPY rescues body weight defects, ameliorates memory deficits and fur loss in Z24-KO mice

**Fig. 2:**
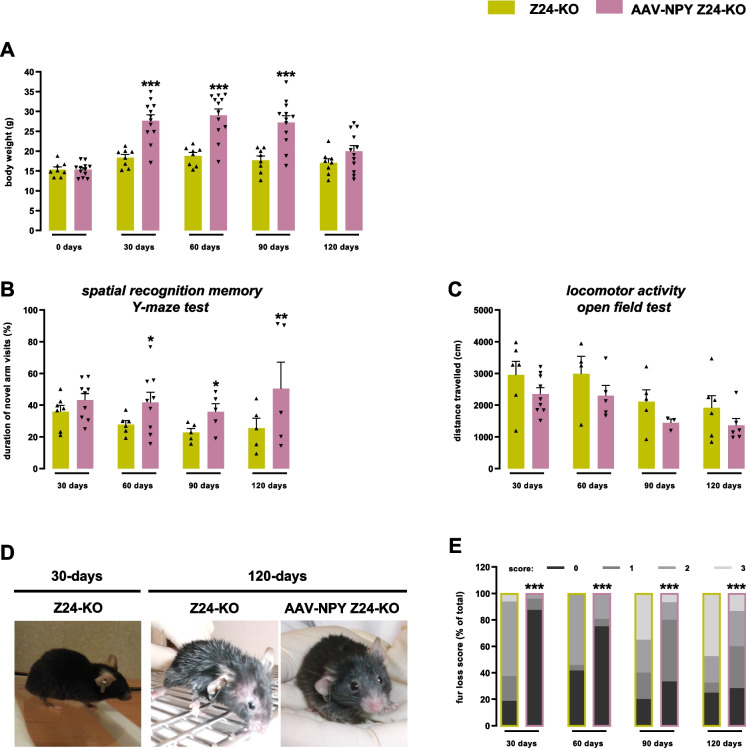
Hypothalamic NPY rescues body weight defects, ameliorates memory deficits and fur loss in Z24-KO mice. **A** AAV-NPY Z24-KO mice exhibit higher weight gain compared to Z24-KO mice. The body weight (g) of male and female mice in both Z24-KO and AAV-NPY Z24-KO groups was monitored at surgery day (day 0) and 30-, 60-, 90-, and 120-days post-injection. **B** and **C** Hypothalamic NPY-AAV injection has no impact on the locomotor activity but enhances memory. AAV-NPY Z24-KO mice display improved duration of visits (%) to novel arms in the Y-Maze test, indicative of enhanced memory function (**B**). Horizontal locomotor activity assessed using total distance traveled (cm) in the open field test shows no differences between groups (**C**). **D** and **E** AAV-NPY Z24-KO show reduced fur loss. Representative photographs captured at 30- and 120-days post-injection, depicting differences in fur condition between Z24-KO and AAV-NPY Z24-KO groups (**D**). Fur loss was evaluated biweekly and scored on a scale of 0 to 3 (0 for absent, 1 for mild, 2 for moderate, and 3 for marked fur loss). Scores were aggregated for each group after over 30-, 60-, 90-, and 120-days post-injection, and frequency distribution was plotted, expressed as a percentage relative to Z24-KO mice (**E**). Results are represented as the mean ± SEM. *p < 0.05, **p < 0.01, ***p < 0.001, determined by Two-way ANOVA, Chi-square test, or Student’s t-test, as appropriate. *N* = 3–12 *per* experimental group. Z24-KO = Zmpste24-KO; AAV = Adeno-Associated Virus; NPY = Neuropeptide Y

Thirty days post-injection of AAV-NPY, AAV-NPY Z24-KO mice exhibited a significant increase in body weight (27.7 ± 0.4 g), compared to Z24-KO mice (18.3 ± 0.8 g, Fig. 2A), maintained up to 90 days post-injection. Since the animals were pair-feed, the increase of body weight cannot be accounted by increase food intake.

We also investigated the impact of hypothalamic AAV-NPY on mouse spatial memory recognition ability using the Y-Maze test. We observed that AAV-NPY Z24-KO mice in the test through the enhanced performance in this test (Fig. 2B). No differences in locomotor activity were observed between groups in the Open-Field test (Fig. 2C). Additionally, we assessed the overall phenotype of Z24-KO mice following hypothalamic AAV-NPY injection, revealing improvements in several health and physical aspects, such as body composition, mobility, and vitality (Fig. 2D). Fur loss, often associated with aging skin and hair follicles, was significantly attenuated in AAV-NPY Z24-KO mice (Fig. 2E).

Overall, these findings suggest that hypothalamic NPY overexpression can ameliorate body weight loss, memory deficits and fur loss in Z24-KO mice, offering insights into potential interventions for age-related phenotype.

### Hypothalamic NPY overexpression reverts lipodystrophy of Z24-KO mice

**Fig. 3:**
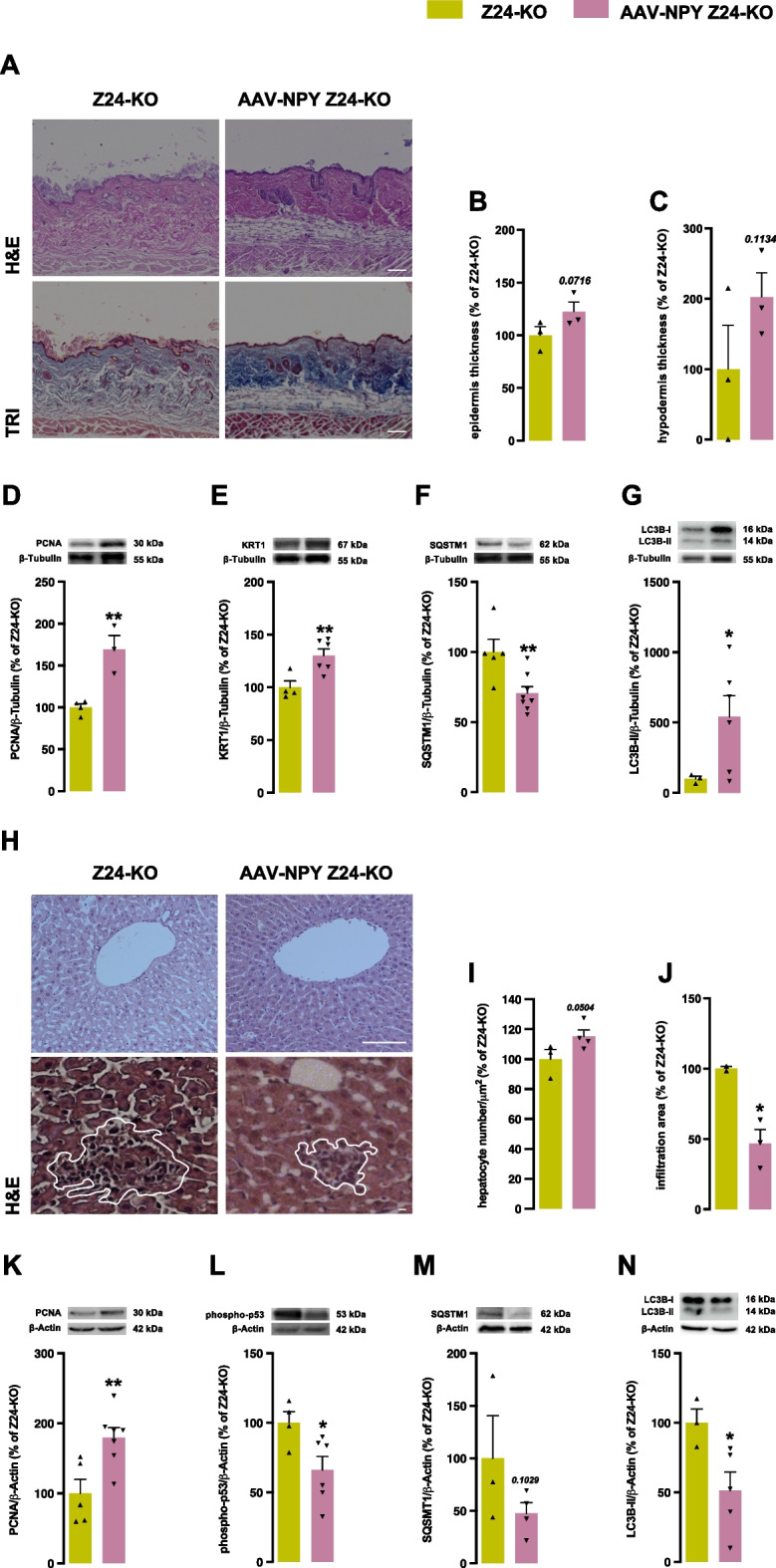
Hypothalamic NPY overexpression reverts lipodystrophy of Z24-KO mice. **A**, **B**, **C**, **D**, **E** and **F** Hypothalamic NPY-overexpression increases thickness of the hypodermis subcutaneous fat layer in the skin. Representative images of dorsal skin sections stained with Haematoxylin–Eosin (top panel) and Masson's-Trichrome (bottom panel) for Z24-KO and AAV-NPY Z24-KO mice. (**A**). Quantification reveals increased epidermis (**B**) and hypodermis subcutaneous fat layer thickness (**C**) (expressed in μm) in the dorsal skin of AAV-NPY Z24-KO mice. Skin lysates collected at the endpoint (four months post-AAV-NPY injection) underwent Western blotting analysis for PCNA (**D**), KRT1 (**E**), SQSTM1 (**F**), and LC3B-II (**G**) as well as and β-Tubulin (loading control). (**H**, **I**, **J**, **K**,** L**, **M** and **N**) NPY-overexpression in the hypothalamus enhances hepatocyte proliferation and reduces immune cell infiltration. Representative Haematoxylin–Eosin-stained liver images for Z24-KO and AAV-NPY Z24-KO mice (**H**). Quantification reveals increased hepatocyte density, expressed in mm^2^ (**I**). Quantification shows reduced infiltration area, indicated by immune cell clusters, expressed in number/μm^2^ (**J**). Liver lysates underwent collected at the endpoint Western blotting analysis for PCNA (**K**), phospho-p53 (**L**), SQSTM1 (**M**), and LC3B-II (**N**) as well as and β-Actin (loading control). Representative Western blot images are shown above the respective graph. Results, represented as mean ± SEM and expressed as a percentage relative to Z24-KO mice. *p < 0.05, **p < 0.01, ***p < 0.001, determined by Student’s *t-*test. Scale bar, 100 μm. *N* = 3–7 *per* experimental group. Z24-KO = Zmpste24-KO; AAV = Adeno-Associated Virus; NPY = Neuropeptide Y

Z24-KO mice exhibit severe lipodystrophy, which was improved upon NPY hypothalamic up-regulation (Supplementary Fig. S2A). This improvement was reflected in the skin, where thicker epidermis and subcutaneous adipose layers suggested amelioration of the lipodystrophic phenotype (Fig. 3A, upper panel, B and C). Furthermore, the skin AAV-NPY Z24-KO mice showed higher collagen (Fig. 3A, bottom panel), PCNA (a cell proliferation marker) (Fig. 3D), and KRT1 levels (a structural marker) (Fig. 3E). These results suggest that hypothalamic NPY might protect skin structure and integrity. To further understand the role of autophagy, we evaluated autophagy markers in the skin. SQSTM1 levels were lower in AAV-NPY Z24-KO mice (Fig. 3F). Higher levels of LC3B-II were also observed (Fig. 3G), which could indicate increased autophagosome formation, leading to enhanced autophagic degradation. These findings suggest increased autophagic activity, potentially reflecting improved cellular health.

The liver, a crucial metabolic organ, undergoes age-related structural and functional changes [35]. In the liver of AAV-NPY Z24-KO mice, we observed an improved liver structure, characterized by smaller hepatocytes, increased hepatocyte number, and reduced immune cell infiltration (Fig. 3G, H and I, respectively). These changes were accompanied by higher PCNA levels, indicative of increased cell proliferation (Fig. 3J). Furthermore, lower phospho-p53 (pp53) levels in AAV-NPY Z24-KO mice suggested a reduction in apoptotic cells (Fig. 3K). Autophagy assessment (Fig. 3M and N) revealed a significant decrease in LC3B-II levels, indicative of enhanced autophagic turnover. This heightened autophagic activity may play a role in maintaining cellular health and supporting liver function.

Concerning heart structure, no apparent differences were observed between Z24-KO and AAV-NPY Z24-KO mice (Supplementary Fig. S2B). However, cardiomyocytes f AAV-NPY Z24-KO mice showed lower cross-sectional area and perimeter, when compared to Z24-KO mice (Supplementary Fig. S2C, D and E), suggesting a potential counteracting effect against myocyte hypertrophy. No differences between groups were observed in kidney structure (Supplementary Fig. S2F), glomerular density was comparable between untreated and AAV-NPY Z24-KO mice (Supplementary Fig. S2G, H and I).

Overall, these findings highlight the multifaceted benefits of hypothalamic NPY in protecting the skin, adipose tissue, and liver of aging.

## Discussion

Aging encompasses a wide array of physiological transformations, including disruptions in neuroendocrine signaling, metabolism and tissue function. In this study, we investigated the relevant role of hypothalamic neuropeptide Y (NPY) in age-related changes using an accelerated aging mouse model, the *Zmpste24*^*−/−*^ (Z24-KO) mice. Our study provides essential insights into the multifaceted benefits of the maintenance of NPY levels within the hypothalamus to prevent age-related changes across various tissues.

Aging promotes a decline in NPY levels in brain regions like the hypothalamus, cortex, and hippocampus [31, 33, 36]. This decrease has been associated with neurodegenerative diseases [37–39]. Conversely, increased NPY levels can mimic the effects of caloric restriction, leading to hyperphagia [40, 41], reduced blood glucose levels [42–44], and lower core body temperature [45, 46] in humans and rats upon central administration. In line of this, the overexpression of NPY in rats has been associated with prolonged lifespan [20]. Prior findings show that caloric restriction fails to extend the lifespan of NPY knockout mice, underscoring the pivotal role of NPY in the regulation of lifespan and the aging process [21]. Female centenarians exhibit elevated blood NPY levels [47], further strengthening its involvement in lifespan. Considering that NPY in the hypothalamus declines with age and that NPY regulates autophagy in the hypothalamus [22], we hypothesized that adjusting NPY levels could potentially generate protective effects against age-related hypothalamic impairments.

The Z24-KO mouse model has been designed to recapitulate human aging. At birth, these mice display typical development but undergo rapid growth retardation post-weaning, experiencing accelerated aging and a premature death at 5 to 6 months of age [25, 48]. Although this mouse model is used as an aging model, age related hypothalamic alterations remained unexplored [9]. Here we show that Z24-KO mice exhibit aged-related changes in the hypothalamus, namely present lower levels of NPY levels in the hypothalamic arcuate nucleus (ARC), compared to wild-type mice. This observation aligns existing literature, reporting age-related NPY decline across diverse brain regions [31, 33]. Considering the relevant role of the hypothalamus in whole-body aging [9], a decline in hypothalamic NPY levels could potentially disrupt this brain region functionality, initiating systemic repercussions. Our previous work demonstrated that upregulating of NPY stimulates autophagy within the rodent hypothalamus [22]. Moreover, NPY plays a crucial role in regulating neuronal function and has been implicated in relevant molecular and cellular of aging processes [49], including neuroprotection and inhibiting neuroinflammation [39, 50]. In addition to autophagy, NPY may interact with other neuroendocrine and metabolic pathways to modulate the aging process in Z24-KO mice. In fact, NPY plays a multifaceted role in the aging process by regulating energy balance and metabolism, impacting key longevity pathways like AMPK and sirtuins, improving stress resilience, providing neuroprotection and modulating [17, 22, 39, 51–53].

Our findings suggest that the age-related decline in NPY levels may contribute to neuronal changes within the ARC, as evidenced by decreased levels of NeuN, a recognized hallmark of neuronal aging. The impact of aging on neuronal density is controversial. While some authors have reported a selective reduction in neuronal density within the aged human brain [54], others contend that such reduction is not a feature of physiological aging [55]. The origin of this controversy might be related to the brain imaging techniques used for these studies, as some methods may possess lower sensitivity when compared to alternative approaches [56]. This decrease in NeuN expression may be attributed to neuronal cell death, reduced proliferation of neuronal progenitor cells, or alterations in neuronal differentiation or activity. Neurogenesis is a natural process by which new cells are generated from a small population of multipotent stem cells in the adult nervous system [57]. There is evidence that neurogenesis decreases with aging in brain regions of mice such as hippocampus [58, 59]. Remarkably, we demonstrate that increasing hypothalamic NPY levels reverses this decline in NeuN expression, emphasizing the neuroprotective role of NPY in maintaining neuronal integrity and mitigating age-associated neuronal changes.

Other brain cells, such as microglia, act as vigilant first responders, upholding CNS homeostasis and serving as an immune surveillance system [60]. Upon detecting threats, microglia undergo a transformation from a resting, ramified morphology to an activated, ameboid state [61]. The protein marker Iba1 is commonly used to study microglia in both quiescent and activated states, facilitating the assessment of cell quantity and morphology (activation) [62–66]. Furthermore, microglial reactivity can trigger astrocyte activation, leading to the release of cytokines and contributing to the preservation of cerebral tissue integrity and neuronal protection [67]. However, while microglial response is typically an initial adaptive response to various insults, it may lead to cytotoxic effects. In aging and age-related conditions, a decreased immune response capacity appears to be associated with neuropathological hallmarks such as beta-amyloid plaques and neurofibrillary tangles [68–71]. This heightened proliferation and reduced immune response capacity are also observed in the brains of aging individuals [72]. In this study, we demonstrate that NPY-AAV leads to a reduction in GFAP levels, indicating a potential modulation of microglia and, consequently, a diminished immune response to neuronal damage, a process commonly dysregulated in aging. Furthermore, our results suggest that AAV-NPY Z24-KO mice have a restored immune response capacity, as indicated by Iba1 levels, highlighting NPY role in inhibiting neuroinflammation. The decreased Iba1 may reflect either reduced activation or a loss of microglial cells, which can have significant implications for immune surveillance and neural homeostasis [73, 74]. This observation may suggest a compromised immune response. These findings underscore the neuroprotective and anti-inflammatory effects of hypothalamic NPY, crucial for maintaining neuronal health during aging.

Others already showed that aging is associated to hypothalamic NF-κB activation, suggesting an age-related hypothalamic neuroinflammation [9]. Therefore, to investigate the beneficial role of hypothalamic NPY preventing neuroinflammation in Z24-KO mice, we assessed the IκBα levels. IκBα is a NF-κB ligand, and it is released upon phosphorylation, initiates NF-κB activation, essential for inflammation [75]. NPY overexpression decreased levels of phosphorylated IκBα (phospho-Iκβα) and Tau, suggesting reduced NF-κB activation in the hypothalamus. These findings suggest a beneficial effect of NPY in alleviating the neuroinflammatory processes observed in the hypothalamus of Z24-KO mice.

Hypothalamic autophagy and NPY levels decrease with aging [18, 23]; here we show that AAV-NPY Z24-KO mice have lower SQSTM1 and LC3B-II protein content, suggesting that NPY regulates autophagy in the hypothalamus. Modulation of NPY levels may be manipulated to yield protective effects against age-related hypothalamic impairments. Furthermore, our study reaffirms our prior findings, demonstrating that the overexpression of hypothalamic NPY induces autophagy [22].

Several age-related histopathological alterations of the Z24-KO mice were previously described by others, highlighting a lipodystrophic phenotype [76]. As expected, we also observed that Z24-KO mice have lower body weight, when compared to age-matched wild type mice. However, after 30 days of NPY-AAV injection, the low body weight of AAV-NPY Z24-KO mice was reverted, reaching the wild-type body weight, maintained up to 90 days post-injection. Regarding the potential factors involved, we would like to emphasize that the maintenance of body weight could indeed be attributed mostly to the augmentation of both adipose tissue and muscle mass in AAV-NPY Z24-KO mice. Interestingly, these beneficial effects extend beyond body weight, as hypothalamic NPY also appears to mitigate other aging-related features, such as alopecia, which has been previously linked to aging and metabolic alterations [77–80]. While we do not possess conclusive evidence concerning bone density, it is noteworthy to consider it as a hypothesis, given that NPY has been shown to regulate bone metabolism and improve bone health [81–83]. The enhanced spatial memory recognition of AAV-NPY Z24-KO mice further underscores its neuroprotective effects, consistent with previous studies implicating NPY in cognitive function [84–87].

As part of the natural aging process, the skin undergoes several alterations such as thinning, loss of vascularization, decreased cellularity in the dermis, and a decline in subcutaneous fat within the hypodermis [88]. These processes are accelerated in mouse models of premature aging, intensifying aging characteristics. The plasticity, expansion capacity, and functionality of adipose tissue depend on the differentiation and proliferation potential of adipose-derived stem cells (ADSCs), giving rise to mature adipocytes; however, the diminished proliferative capacity of ADSCs may be intricately associated with the senescent and metabolic changes observed during the natural aging process (reviewed in ref. [89]). We show that hypothalamic NPY promotes thickening of the epidermis. Age-associated skin changes also include the decrease in extracellular matrix components, such as collagen [88]. Hypothalamic NPY increased collagen deposition and KRT1 levels, enhancing skin structure and integrity. Additionally, decreased SQSTM1 and increased LC3B-II levels, indicative of enhanced autophagy in the skin, collectively suggest that hypothalamic NPY and autophagy play a role in improving cellular health and protein turnover, thereby promoting enhanced cellular health, protein turnover, and cell proliferation. The thickening of the subcutaneous fat layer observed in lipodystrophy may result from increased adipocyte proliferation and differentiation, as indicated by elevated levels of PCNA. Importantly, the deficiency in adipose tissue content, a characteristic of the lipodystrophic phenotype, was reversed by hypothalamic NPY, emphasizing a potential avenue for improving lipodystrophy. Restoring adipose tissue and enhancing metabolic equilibrium in individuals with lipodystrophy show promise in mitigating premature aging, indicating potential avenues for therapeutic interventions. Although, in the present study, we induced a twofold increase of hypothalamic NPY, which is similar to what occurs in caloric restriction [90] we recognize that overstimulation of hypothalamic NPY signaling could induce unintended consequences, as hyperphagia and adiposity. These observations underscore the importance of precise modulation of hypothalamic NPY levels to achieve therapeutic benefits while minimizing adverse effects.

The liver, a major metabolic organ, also undergoes structural and functional changes during aging. The hypothalamus assumes a vital role in governing liver functions via neural and neuroendocrine connections, as underscored by its participation in regulating liver metabolism through these pathways [91]. Furthermore, the integrated nature of these pathways, with the hypothalamus serving as a central regulator of hepatic glucose and lipid metabolism [92], goes beyond identifying specific neuronal subpopulations in the hypothalamus that project to the liver, further elucidating the complex neural circuitry involved in this regulation [93]. Hypothalamic NPY improved Z24-KO mice liver structure, characterized by smaller hepatocytes, increased hepatocyte count, and reduced immune cell infiltration. We observed higher PCNA levels in the liver in AAV-NPY Z24-KO mice suggestive of increased cell proliferation, potentially contributing to liver protection or regeneration. Our results indicate a decrease in LC3B-II, suggesting that hypothalamic NPY overexpression may induce autophagy in the liver, as others have previously demonstrated that activation of NPY neurons in ARC induces autophagy in the liver [94]. Taken together, these findings suggest that the hypothalamic NPY plays a role in protecting liver integrity and function.

In conclusion, the present study shows that reestablishing hypothalamic NPY levels mitigate Z24-KO mice aging phenotype. However, long-term studies are needed to fully understand whether these observed effects translate into healthy lifespan of treated animals. Further studies are needed to better understand the potential of translational approach of restoring hypothalamic NPY levels to delay aging phenotypes and frailty of age-related conditions.

## Supplementary Information

Below is the link to the electronic supplementary material.

Supplementary file1 (DOCX 7871 KB)

## Data Availability

All data originated in this study is included in the manuscript and Supplementary information. Additional information is available from the corresponding authors upon reasonable request.

## Abbreviations

AAV: Adeno-Associated Virus
ADSCs: Adipose-Derived Stem Cells
ARC: Arcuate Nucleus
GFAP: Glial Fibrillary Acidic Protein
GFAP: Glial Fibrillary Acidic Protein
GH: Growth Hormone
HGPS: Hutchinson-Gilford progeria syndrome
Iba1: Ionized calcium-binding adaptor molecule 1
IKKβ: Inhibitory kappa B kinase beta
KO: Knockout
KRT1: Keratin 1
LC3B-II: Microtubule-associated protein 1A/1B-light chain 3
NeuN: Neuronal Nuclear Protein
NF-κB: Nuclear factor kappa B
NPY: Neuropeptide Y (NPY)
PCNA: Proliferating Cell Nuclear Antigen
SQSTM1: Sequestosome 1
Z24: Zmpste24
