# Supplementary material for: Restoring neuropetide Y levels in the hypothalamus ameliorates premature aging phenotype in mice
Source: GeroScience. 2025 Feb 27;47(4):5785–801. doi: 10.1007/s11357-025-01574-0 (PMC12397475; doi:10.1007/s11357-025-01574-0)
Supplement: Supplementary file 1 — Supplementary file1 (DOCX 7871 KB) [file 11357_2025_1574_MOESM1_ESM.docx]

***Supporting Information for***

***RESTORING NEUROPETIDE Y LEVELS IN THE HYPOTHALAMUS AMELIORATES PREMATURE AGING PHENOTYPE IN MICE***

*Short running title:* ***Hypothalamic Neuropeptide Y Reverses Premature Aging in Mice***

Marisa Ferreira-Marques, MSc^1,2,11,*^, Sara Carmo-Silva, PhD^3,4,*^, Joana Pereira, MSc^1,2,11^, Mariana Botelho, PhD^1,2,11^, Clévio Nóbrega, PhD^5,6^, Carlos López‐Otín, PhD^7,8,9^, Luís Pereira de Almeida, PhD^1,2,11^, Célia A. Aveleira, PhD^1,2,10,^**^#^**, Cláudia Cavadas, PhD^1,2,11,^**^#^**

^1^CNC-UC, Center for Neuroscience and Cell Biology, University of Coimbra, Coimbra, Portugal;

^2^CIBB - Center for Innovative Biomedicine and Biotechnology, University of Coimbra, Coimbra, Portugal;

^3^H&TRC – Health and Technology Research Center, Coimbra Health School, Polytechnic University of Coimbra, Coimbra, Portugal;

^4^ciTechCare—Center for Innovative Care and Health Technology, Polytechnic University of Leiria, Leiria, Portugal;

^5^Algarve Biomedical Center Research Institute (ABC-RI), University of Algarve, Faro, Portugal;

^6^Faculty of Medicine and Biomedical Sciences (FMCB), University of Algarve, Faro, Portugal;

^7^Departamento de Bioquímica y Biología Molecular, Facultad de Medicina, Instituto Universitario de Oncología, Universidad de Oviedo, Oviedo, Spain;

^8^Centre de Recherche des Cordeliers, Inserm U1138, Sorbonne Université, Paris, France;

^9^Facultad de Ciencias de la Vida y la Naturaleza, Universidad Nebrija, Madrid, Spain;

^10^MIA-Portugal - Multidisciplinar Institute of Ageing, University of Coimbra, Coimbra, Portugal;

^11^Faculty of Pharmacy, University of Coimbra, Coimbra, Portugal;

^*^equal contribution;

**^#^**equal senior contribution;

***Correspondence:*** Cláudia Cavadas, ^1^CNC-UC, Center for Neuroscience and Cell Biology, University of Coimbra, Coimbra, Portugal [ccavadas@uc.pt](mailto:ccavadas@uc.pt)

***Classification:*** Research Articles

***This PDF file includes:***

Figures S1 to S2

***
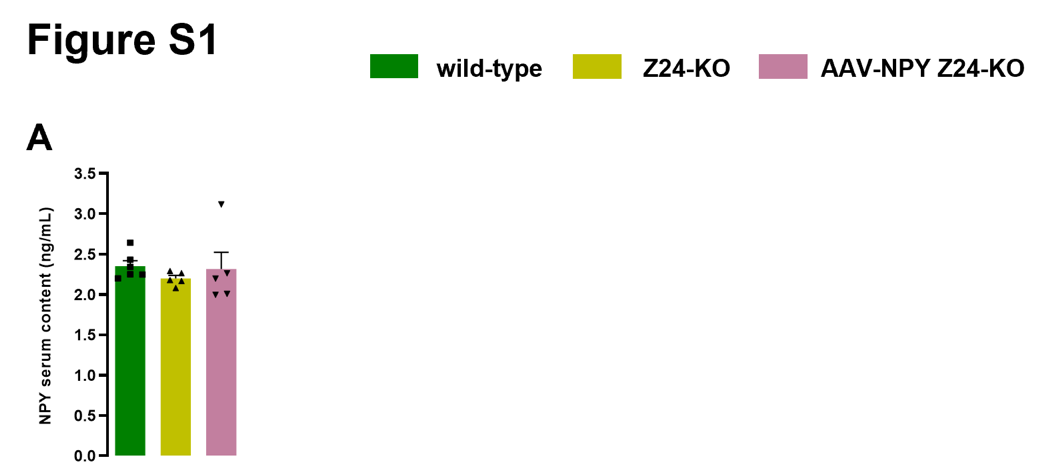
***

***Figure S1. NPY-AAV in the hypothalamus had no effect on circulating levels of serum NPY in Z24-KO mice.*** (**A**) Hypothalamic NPY overexpression showed no impact on circulating serum NPY levels in Z24-KO mice. Serum NPY content is expressed in ng/mL. Data are expressed as the mean±SEM. *N*=5-6 *per* group. Z24-KO = Zmpste24-KO; AAV= Adeno-Associated Virus; NPY = Neuropeptide Y.


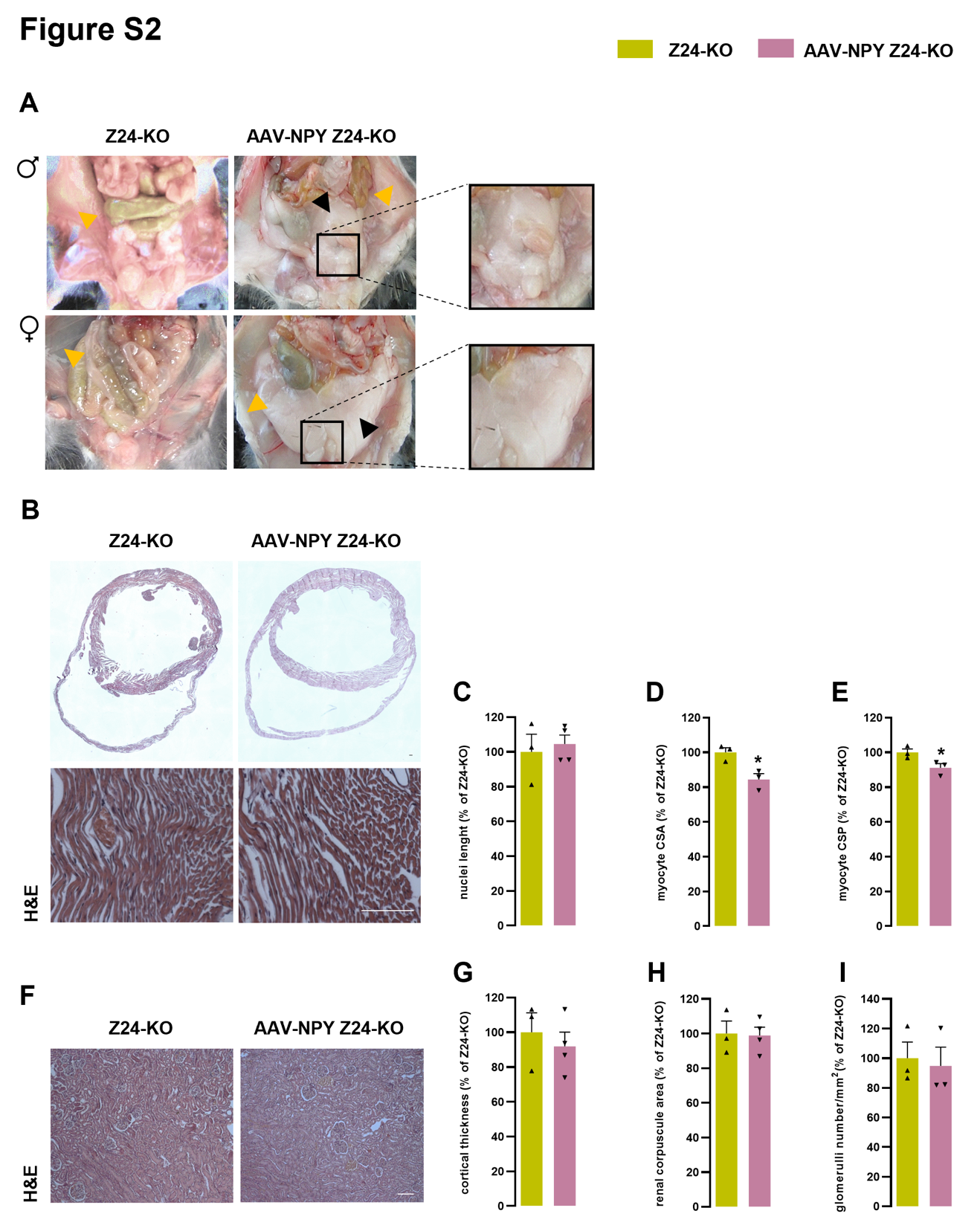


***Figure S2. Hypothalamic NPY increases adipose tissue deposition in Z24-KO mice.*** (**A**) Noticeably enhanced adipose tissue depots in male and female Z24-KO mice following hypothalamic NPY-overexpression. AAV-NPY Z24-KO mice display significantly larger adipose depots, including subcutaneous and gonadal adipose tissue. Representative images, taken 120 days after injection, illustrate AAV-NPY Z24-KO mice with subcutaneous (yellow arrows) and visceral (black arrows) fat depots in comparison to Z24-KO mice. (**B**, **C**, **D**, and **E**) Impact of hypothalamic NPY modulation on the heart of Z24-KO mice. Representative images of Haematoxylin-Eosin-stained cross-sectional paraffin slices of the heart for Z24-KO and AAV-NPY Z24-KO mice (**B**). Quantification of the nuclear length of the cardiomyocyte longitudinal fibers, expressed in μm (**C**). Quantification of cross-sectional area and perimeter of the cardiomyocyte fibers, normalized to Z24-KO mice group (**D** and **E**, respectively). (**F**, **G**, **H**, and **I**) Influence of hypothalamic NPY-overexpression on the histopathological structure of the liver. Representative images of Haematoxylin-Eosin-stained longitudinal paraffin slices of the kidney for Z24-KO and AAV-NPY Z24-KO mice (**F**). Quantification of cortical thickness in longitudinal kidney sections, expressed in μm (**G**). Quantification of renal corpuscle area and glomeruli density, expressed in μm^2^ and *per* mm^2^ (**H** and **I**, respectively). Results, represented as mean±SEM. **p*<.05 determined by Student’s *t-*test. Scale bar, 100 μm. *N*=3-4 *per* experimental group. Z24-KO = Zmpste24-KO; AAV= Adeno-Associated Virus; NPY = Neuropeptide Y.
